# A Prognostic Ferroptosis-Related lncRNA Model Associated With Immune Infiltration in Colon Cancer

**DOI:** 10.3389/fgene.2022.934196

**Published:** 2022-08-31

**Authors:** Jianzhong Lu, Jinhua Tan, Xiaoqing Yu

**Affiliations:** School of Science, Shanghai Institute of Technology, Shanghai, China

**Keywords:** colon cancer, ferroptosis, long non-coding RNA, prognostic model, immune microenvironment

## Abstract

Colon cancer (CC) is a common malignant tumor worldwide, and ferroptosis plays a vital role in the pathology and progression of CC. Effective prognostic tools are required to guide clinical decision-making in CC. In our study, gene expression and clinical data of CC were downloaded from The Cancer Genome Atlas (TCGA) and Gene Expression Omnibus (GEO) databases. We identified the differentially expressed ferroptosis-related lncRNAs using the differential expression and gene co-expression analysis. Then, univariate and multivariate Cox regression analyses were used to identify the effective ferroptosis-related lncRNAs for constructing the prognostic model for CC. Gene set enrichment analysis (GSEA) was conducted to explore the functional enrichment analysis. CIBERSORT and single-sample GSEA were performed to investigate the association between our model and the immune microenvironment. Finally, three ferroptosis-related lncRNAs (XXbac-B476C20.9, TP73-AS1, and SNHG15) were identified to construct the prognostic model. The results of the validation showed that our model was effective in predicting the prognosis of CC patients, which also was an independent prognostic factor for CC. The GSEA analysis showed that several ferroptosis-related pathways were significantly enriched in the low-risk group. Immune infiltration analysis suggested that the level of immune cell infiltration was significantly higher in the high-risk group than that in the low-risk group. In summary, we established a prognostic model based on the ferroptosis-related lncRNAs, which could provide clinical guidance for future laboratory and clinical research on CC.

## Introduction

Colon cancer (CC) has the third most incidences among malignancies, and it is the second most common cause of cancer death in men and women combined (Siegel et al., 2022). The malignant transformation of CC is a multistep process that takes approximately ten years from small clumps to CC (Jemal et al., 2011). Therefore, early diagnosis is essential for improving the prognosis of CC patients. However, the survival of CC patients is poor because of the complexity of the disease, late disease detection, and lack of reliable risk-assessment biomarkers (Lin et al., 2020; Yang C. et al., 2021). Even after treatment, the risk of recurrence and metastasis in CC patients is still high (Chang et al., 2020; Jin et al., 2020). In recent years, more studies have suggested that it is promising to solve the problem by integrating computational techniques with big biomedical data involving multiple types of biomarkers including epigenetic, genetic, and gene expression profiles (Yang Y. et al., 2021; Liu et al., 2021). Therefore, identifying effective biomarkers to establish a prognostic model for survival prediction is gaining increasing attention.

lncRNAs are non-protein coding transcripts over 200 nucleotides in length (Mercer et al., 2009). There are more than 50,000 lncRNA genes annotated in the human genome (Borkiewicz et al., 2021). Studies have shown that lncRNAs are often dysregulated during tumorigenesis, which might cause tumor development (Prensner and Chinnaiyan, 2011; Schmitt and Chang, 2016). Therefore, they are used as molecular biomarkers to diagnose and treat many diseases, including CC. For example, Zhou et al. (2019) revealed that lncRNA XIRP2-AS1 has a favorable impact on the overall survival of patients with colon cancer. Tsai et al. (2018) found that lncRNA Linc00659 expression knockdown could accelerate cell apoptosis in CC cells treated with chemotherapy drugs.

Ferroptosis is a newly discovered form of programmed cell death characterized by iron-dependent accumulation of lethal lipid peroxidation (Tang et al., 2018; Mou et al., 2019). Cancer cells are vulnerable to ferroptosis because of their high iron uptake to support fast proliferation (Hassannia et al., 2019). Recently, studies have demonstrated that ferroptosis plays a crucial role in tumorigenesis and cancer therapeutics. Wang et al. (2021) constructed a ferroptosis-related prognostic signature for LUAD and suggested that ferroptosis is a functional and therapeutic target in LUAD. He et al. (2021) have constructed a prognostic risk model based on 10 genes related to ferroptosis and identified potential novel therapeutic targets which improve the individualized treatment of patients with HNSCC. Moreover, considering the critical role of ferroptosis in cancer, many studies proposed ferroptosis-based strategies to identify potential lncRNA biomarkers associated with various cancers. For example, Guo et al. (2021) revealed that ferroptosis-related lncRNAs have the potential to inform immunological research and treatment. Wei et al. (2021) identified that ferroptosis-related lncRNAs have an important prognostic value in gastric cancer. Feng et al. (2022) suggested that ferroptosis and iron metabolism–related lncRNAs can independently predict the overall survival and therapeutic effect in patients with ovarian cancer. Currently, many prognostic models have been proposed based on the ferroptosis-related lncRNAs for colon cancer (Cai et al., 2021; Zhang et al., 2021). However, the functional mechanisms of the ferroptosis-related lncRNAs and the relationship between the prognostic model and the tumor immune microenvironment require further investigation for CC patients.

**FIGURE 1:**
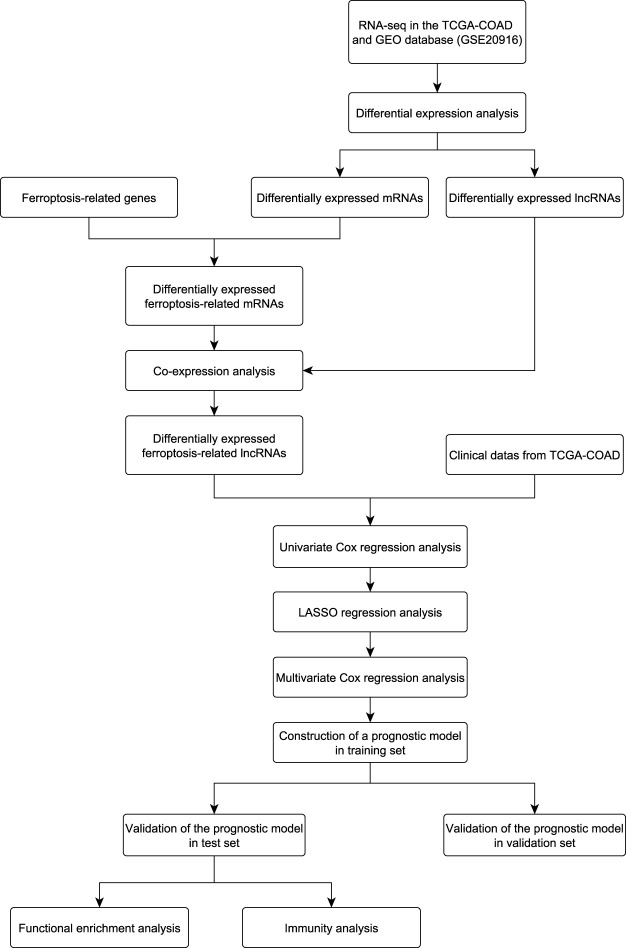
Flowchart of this study.

In this study, three ferroptosis-related lncRNAs were identified as the prognostic biomarkers for CC. The prognostic model based on the ferroptosis-related lncRNAs was constructed for predicting the overall survival of CC patients, which would provide prognostic insights into anticancer therapies and a novel source for immune therapies. The workflow of this study is shown in Figure 1.

## Materials and Methods

### Data Collection

In this study, we selected four independent datasets from two different high-throughput platforms, including 458 colon adenocarcinoma (COAD) samples and 41 normal samples from TCGA (https://portal.gdc.cancer.gov/); 111 CC samples, 34 normal samples (GSE20916), 124 colorectal cancer samples (GSE72970), and 177 CC samples (GSE17536) from the GEO (https://www.ncbi.nlm.nih.gov/geo/). The gene expression profiling of the three datasets (GSE20916, GSE72970, and GSE17536) was based on the GPL570 platform. Patients with a survival time of more than 30 days were used for the survival analysis. The detailed clinical characteristics of the patients are shown in Table 1. We downloaded 259 ferroptosis-related genes from the FerrDb database (Zhou and Bao, 2020), including 108 driver genes, 69 suppressor genes, and 111 marker genes (Supplementary Table S1).

**TABLE 1:** Characteristics of CC patients in our study.

Characteristic	Training set (*n* = 185)	Test set (*n* = 185)	GSE72970 (*n* = 124)	GSE17536 (*n* = 177)
Age (years)				
<70	96	107	90	104
≥70	89	78	34	73
Gender				
Female	85	86	50	81
Male	100	99	74	96
T stage				
T1	5	4	1	—
T2	32	33	7	—
T3	134	121	50	—
T4	14	27	37	—
TX	—	—	29	—
N stage (pN)				
N0	118	101	14	—
N1	36	51	28	—
N2	31	33	53	—
NX	—	—	29	—
M stage				
M0	160	150	22	—
M1	25	35	102	—
TNM stage				
I	33	32	0	24
II	82	63	6	57
III	45	55	15	57
IV	25	35	102	39
X	—	—	1	—

### Identification of Differentially Expressed Ferroptosis-Related lncRNAs

In this study, we identified mRNAs and lncRNAs using the Ensembl database (http://ensemblgenomes.org). The expression profile of mRNAs and lncRNAs was extracted from RNA-seq count data, which was normalized using the edgeR package (version 3.32.1). Differentially expressed mRNAs and lncRNAs shared by TCGA-COAD and GSE20916 were identified using the edgeR and limma R packages [ $\left|\log_{2}\left(FoldChange\right)\right|>1$ and p<0.05 ]. The intersection between the differentially expressed mRNAs (DEmRNAs) and the 259 ferroptosis-related genes was defined as differentially expressed ferroptosis-related mRNAs (DEFR-mRNAs). We constructed the co-expression network with the DEFR-mRNAs and the differentially expressed lncRNAs (DElncRNAs) based on the Pearson correlation analysis to identify the differentially expressed ferroptosis-related lncRNAs (DEFR-lncRNAs). In the co-expression network, the DElncRNAs with $\left|R^{2}\right|>0.4$ and p<0.001 remained as the DEFR-lncRNAs.

### Construction of a DEFR-lncRNA Prognostic Model

Univariate Cox regression analysis was first performed by integrating the gene expression matrix of the DEFR-lncRNAs and the survival data in TCGA-COAD to identify the DEFR-lncRNAs with prognostic relevance for the overall survival (OS). Statistically significant value was set at p<0.05 . Moreover, the least absolute shrinkage and selection operator (LASSO) regression analysis was used to avoid overfitting and build a reliable and robust model. Next, the screened DEFR-lncRNAs were validated using the multivariate Cox regression analysis, and the DEFR-lncRNAs associated with the prognosis of CC were obtained. Finally, the prognostic risk score (RS) model was constructed for each patient, which was calculated as follows:

$$\mathrm{RS}=\overset{n}{\underset{i=1}{\sum }}\left[expr\left(lncRNA_{i}\right)\times coef\left(lncRNA_{i}\right)\right],$$

where $expr\left(lncRNA_{i}\right)$ is the gene expression value of $lncRNA_{i}$ , and $coef\left(lncRNA_{i}\right)$ is the corresponding estimated regression coefficient in the multivariate Cox regression analysis.

### Enrichment Analysis

Gene set enrichment analysis (GSEA) (http://www.broad.mit.edu/gsea/) is a computational method used to identify whether a pre-defined set of genes shows significant differences between two biological states (Subramanian et al., 2005). GSEA was performed by GSEA software (version 4.2.3). The Kyoto Encyclopedia of Genes and Genomes (KEGG) pathway and Hallmark pathways were used to explore the potential pathways and gene sets associated with the model. They were visualized using the ggplot2 R package.

### Immunity Analysis

CIBERSORT (https://cibersort.stanford.edu/) is an established computational resource to estimate the abundance of member cell types in a mixed cell population (Newman et al., 2015). In our study, we applied the CIBERSORT algorithm to assess the tumor infiltration levels of 22 immune cell types from the CC patients in TCGA-COAD. It was run using the LM22 signature with 1,000 permutations to estimate the relative fractions of the 22 immune cell types. Moreover, the single-sample gene set enrichment analysis (ssGSEA) was also performed, and 28 immune cell types that are over-represented in the tumor microenvironment were analyzed to understand the association between the prognostic model and immune infiltration (Charoentong et al., 2017).

### Statistical Analysis

All statistical analyses were conducted by R software (Version 4.0.2). Univariate Cox regression analysis, LASSO regression analysis, and multivariate Cox regression analysis were performed to identify the DEFR-lncRNAs associated with the prognosis of CC patients. The Kaplan–Meier survival analysis and log-rank test were used to conduct survival analysis. The timeROC R package was used to draw receiver operating characteristic (ROC) curves and quantify the area under the curve (AUC) values. The GSVA R package was used for the ssGSEA.

## Results

### Identification of Differentially Expressed Ferroptosis-Related lncRNAs

**FIGURE 2:**
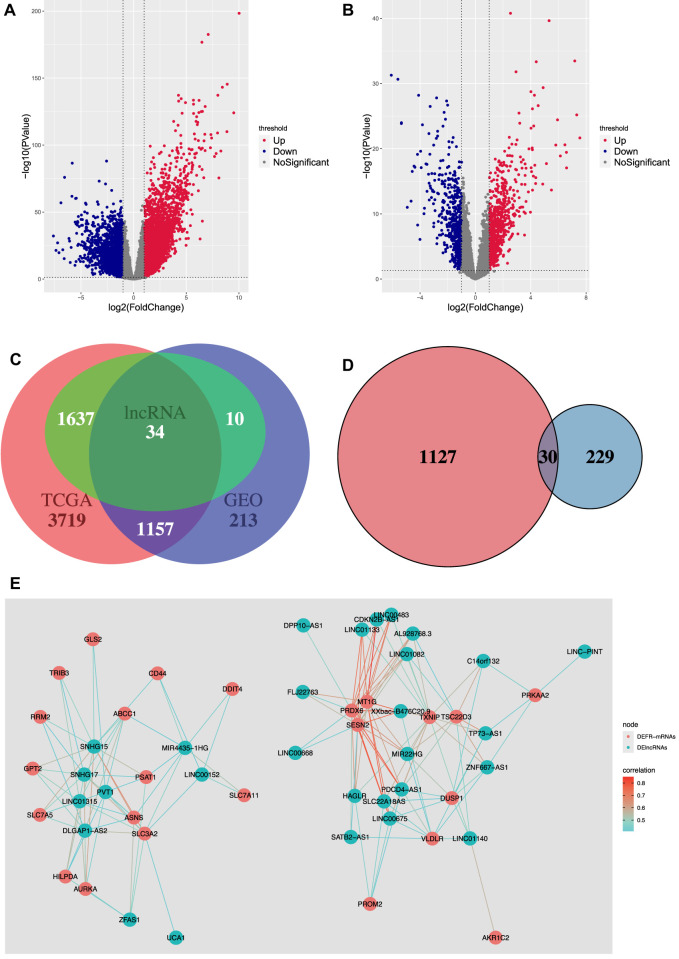
Identification of DEFR-lncRNAs. **(A)** Volcano plot of DEmRNAs and DElncRNAs in TCGA-COAD. **(B)** Volcano plot of DEmRNAs and DElncRNAs in GSE20916. **(C)** Venn diagram of DEmRNAs and DElncRNAs in TCGA-COAD and GSE20916. **(D)** Venn diagram of the shared DEmRNAs and ferroptosis-related genes. Red represents the shared DEmRNAs between TCGA-COAD and GSE20916, and blue represents the ferroptosis-related genes. **(E)** Co-expression network between DEFR-mRNAs and DElncRNAs.

In our study, using the gene type data reported for the genome GRCh38.p13, 19,674 mRNAs and 14,826 lncRNAs were downloaded from TCGA-COAD, and 12,001 mRNAs and 370 lncRNAs were downloaded from GSE20916. The differential expression analysis showed that 4,876 mRNAs and 1,671 lncRNAs were differentially expressed in TCGA-COAD, and 1,370 mRNAs and 44 lncRNAs were differentially expressed in GSE20916. The volcano plots of DEmRNAs and DElncRNAs of TCGA-COAD and GSE20916 are shown in Figures 2A,B, respectively. Moreover, 1,157 DEmRNAs and 34 DElncRNAs shared by the two databases were obtained (Figure 2C). Then, 30 DEFR-mRNAs were obtained after intersecting 1,157 DEmRNAs and 259 ferroptosis-related genes (Figure 2D). Finally, 29 DEFR-lncRNAs were identified using the co-expression analysis, which was shown in the co-expression network (Figure 2E **)**.

### Construction of a Prognostic Model Based on DEFR-lncRNAs

**FIGURE 3:**
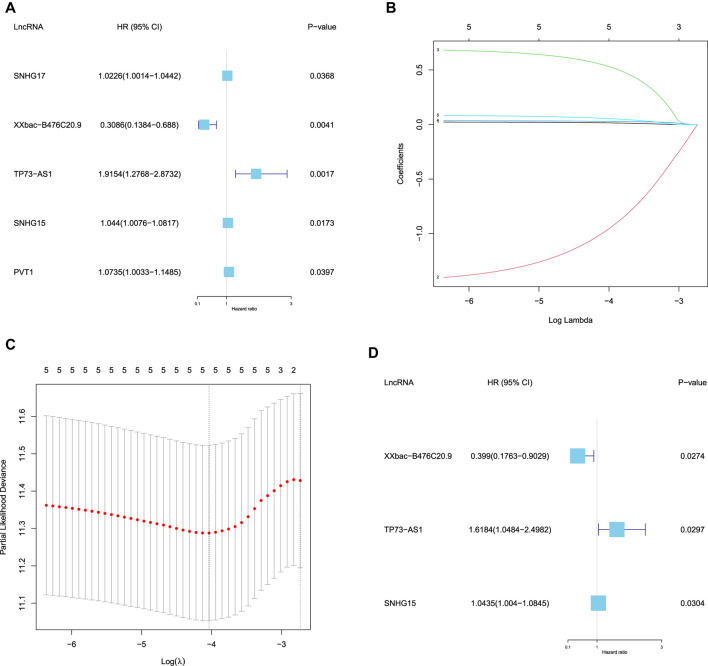
Identification of the prognostic DEFR-lncRNAs. **(A)** Forest map of five DEFR-lncRNAs identified by univariate Cox regression analysis. **(B)** LASSO coefficient profile of the five DEFR-lncRNAs. **(C)** Plots of the cross-validation error rates. **(D)** Forest map of three DEFR-lncRNAs identified by the multivariate Cox regression analysis.

Based on the 29 DEFR-lncRNAs, we identified five DEFR-lncRNAs (SNHG17, XXbac-B476C20.9, TP73-AS1, SNHG15, and PVT1) that were statistically related to the OS of CC patients using the univariate Cox regression analysis ( p<0.05 , Figure 3A). Then, the five DEFR-lncRNAs were subjected to the LASSO regression analysis. As the values of λ increased, the LASSO coefficients of these five lncRNAs decreased to zero (Figure 3B). Moreover, the partial likelihood deviances of different numbers of lncRNAs were revealed by the LASSO regression model, which showed that the model had an optimal performance with the least parameters when log(λ)=−4.035622 (Figure 3C). Subsequently, the multivariate Cox regression analysis was performed, and three DEFR-lncRNAs (XXbac-B476C20.9, TP73-AS1, and SNHG15) were selected as the prognostic DEFR-lncRNAs for constructing the prognostic model ( p<0.05 , Figure 3D).

**FIGURE 4:**
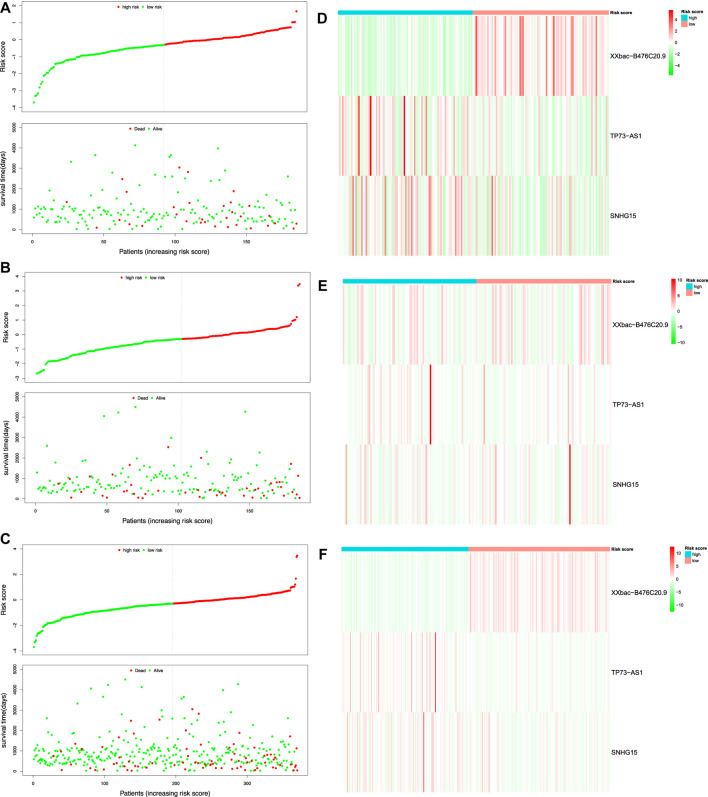
Risk score analysis of the prognostic model in TCGA-COAD. Risk score distribution and survival status of the patients in the training set **(A)**, test set **(B)**, and whole set **(C)**. Expression heatmap of three DEFR-lncRNAs in the training set **(D)**, test set **(E)**, and whole set **(F)**.

After filtering patients with incomplete gene expression data and clinical information, 370 patients in TCGA-COAD remained in our study, who were divided randomly into the training set and the test set in a 1:1 ratio. The prognostic model was constructed based on the three prognostic DEFR-lncRNAs in the training set. The RS was calculated for each patient using the following equation: RS=−2.1053×expr(XXbac−B476C20.9)+0.6008×expr(TP73−AS1)+0.0873×expr(SNHG15) . Patients were classified into high-risk and low-risk groups in the training, test, and whole sets. The cutoff values for the three datasets were the median RS in the training set ( RS=−0.291257 ). We observed that the proportion of patients with CC in the high-risk group was significantly higher than that of the low-risk group in the training, test, and whole sets, respectively (Figures 4A–C). We also investigated the expression of the three prognostic DEFR-lncRNAs in the high-risk and low-risk groups (Figures 4D,E). In the whole set, we can find that the lncRNA XXbac-B476C20.9 was higher expressed in the low-risk group, while the lncRNAs TP73-AS1 and SNHG15 were higher expressed in the high-risk group (Figure 4F).

**FIGURE 5:**
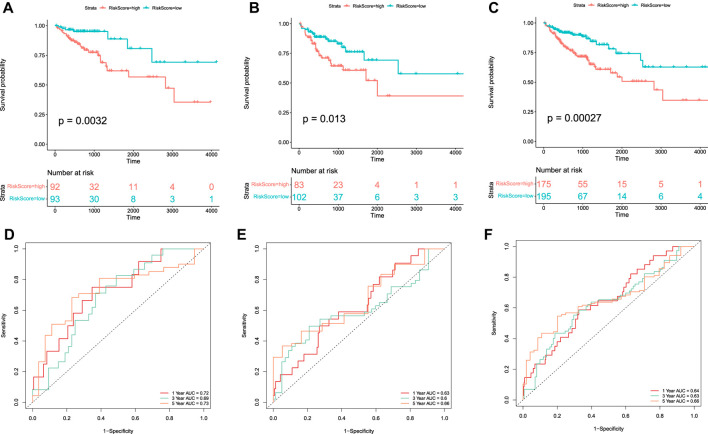
Kaplan–Meier curve and ROC curve of the model in TCGA-COAD. Kaplan–Meier curves of the OS of patients between high-risk and low-risk groups in the training set **(A)**, test set **(B)**, and whole set **(C)**. Time-dependent ROC curve analysis of the RS in the training set **(D)**, test set **(E)**, and whole set **(F)**.

Kaplan–Meier survival curves were plotted to compare the difference in the OS between the high-risk and low-risk groups, which indicated that the patients in the low-risk group had better OS than those in the high-risk group in the training, test, and whole sets (Figures 5A–C). Moreover, time-dependent ROC curves were plotted to assess the sensitivity and specificity of the 1-, 3-, and 5-year survival predictions of CC patients using the timeROC R package. In the training set, the AUCs used for 1-, 3-, and 5-year OS predictions were 0.72, 0.69, and 0.73, respectively (Figure 5D). In the test set, the AUCs used for 1-, 3-, and 5-year OS predictions were 0.63, 0.6, and 0.66, respectively (Figure 5E). In the whole set, the AUCs used for 1-, 3-, and 5-year OS predictions were 0.64, 0.63, and 0.66, respectively (Figure 5F).

**FIGURE 6:**
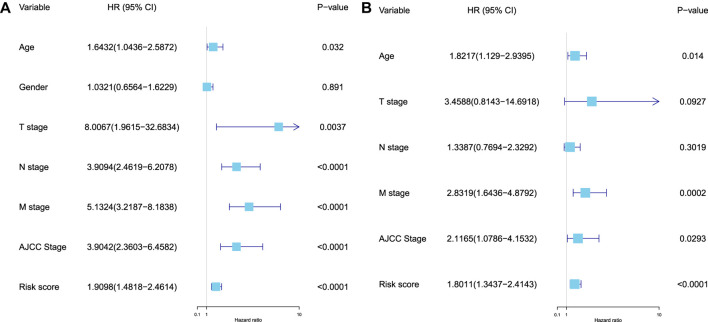
Validation of the independent predictive power of the model. **(A)** Univariate Cox regression analysis of the prognostic variables in the whole set. **(B)** Multivariate Cox regression analysis of the prognostic variables (age, T stage, N stage, M stage, AJCC stage, and RS) in the whole set.

Furthermore, the univariate and multivariate Cox regression analyses were performed to validate the independent predictive power of the prognostic RS model for CC patients in the training, test, and whole sets, and the variables (age, gender, T stage, N stage, M stage, AJCC stage, and RS) were used as the possible risk factors. These results revealed that the prognostic model proposed in our study can be used as an independent prognostic factor for CC patients (Supplementary Table S2). In the whole set, we found that age, M stage, AJCC stage, and RS were the independent risk factors for CC patients ( p<0.05 , Figures 6A,B).

### Verification of the Prognostic Model in the Validation Set

**FIGURE 7:**
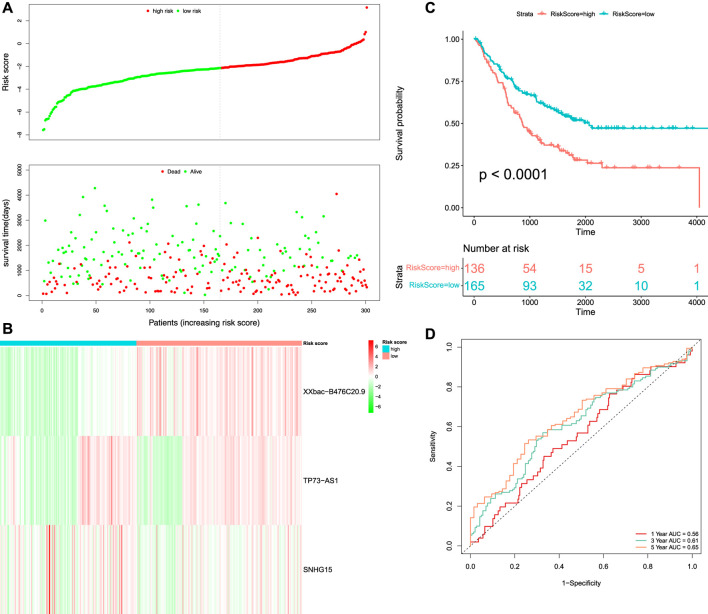
Survival and time-dependent ROC curve analysis of the prognostic model in the validation set. **(A)** Risk score distribution and survival status of the patients. **(B)** Heatmap of three DEFR-lncRNAs. **(C)** Kaplan–Meier curve analysis of the OS between high-risk and low-risk groups. **(D)** Time-dependent ROC curve analysis of the RS.

We merged GSE72970 and GSE17536 to form the validation set, which contained 301 tumor samples. We calculated the RS of each patient in the validation set based on the formula of the prognostic RS model. The patients in the validation set were classified into high-risk (*n* = 136) and low-risk groups (*n* = 165) according to the optimal cut-off value ( RS=−2.150814 ). The distribution of the RS for each patient and their survival status in the validation set are shown in Figure 7A. The death status of the patients increased with the increasing risk score. The expression pattern of the three prognostic DEFR-lncRNAs between the high-risk and low-risk groups is shown as a heatmap in Figure 7B. The Kaplan–Meier survival analysis demonstrated that the patients in the high-risk group had a significantly shorter OS than those in the low-risk group ( p<0.0001 , Figure 7C). The AUC values for the 1-, 3-, and 5-year OS in the validation set were 0.56, 0.61, and 0.65, respectively (Figure 7D).

### Functional Enrichment Analysis

**FIGURE 8:**
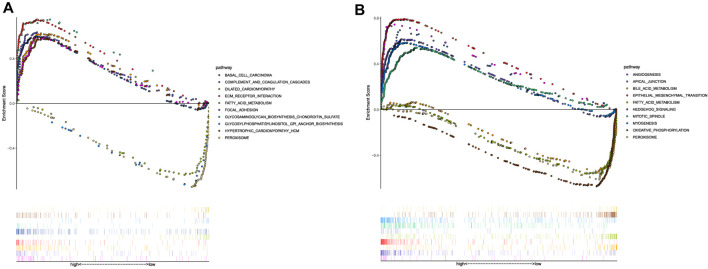
Functional enrichment analysis. **(A)** KEGG pathways with the top 10 NES. **(B)** Hallmark pathways with the top 10 NES.

The GSEA was performed to investigate the potential pathways and functions connected with high-risk and low-risk groups, and the terms p<0.05 and FDR<0.25 were considered statistically significant. The KEGG pathway analysis showed that peroxisome, glycosylphosphatidylinositol (GPI) anchor biosynthesis, and fatty acid metabolism were enriched in the low-risk group, whereas the extracellular matrix (ECM) receptor interaction, dilated cardiomyopathy, focal adhesion, complement and coagulation cascades, hypertrophic cardiomyopathy (HCM), glycosaminoglycan biosynthesis chondroitin sulfate, and basal cell carcinoma were enriched in the high-risk group (Figure 8A). Moreover, the Hallmark pathway analysis also revealed that the high-risk group was mainly enriched for epithelial-mesenchymal transition, apical junction, angiogenesis, hedgehog signaling, myogenesis, and mitotic spindle, whereas the low-risk group was mainly enriched for peroxisome, bile acid metabolism, fatty acid metabolism, and oxidative phosphorylation (Figure 8B). Of note, peroxisomes, fatty acid metabolism, and oxidative phosphorylation enriched in the low-risk group were associated with ferroptosis, which have been reported to be closely linked to ferroptosis (Stockwell et al., 2017; Tang and Kroemer, 2020; Ma et al., 2021).

### Immune Infiltration Analysis

**FIGURE 9:**
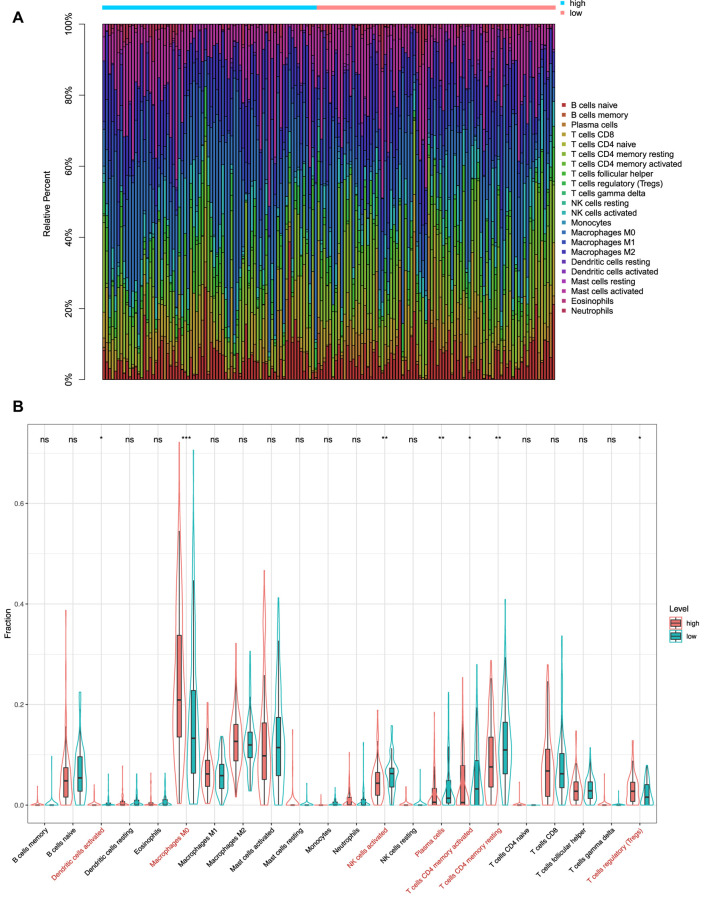
Immunity analysis *via* CIBERSORT. **(A)** Bar graph showing the proportion of 22 immune cell types in CC patients of TCGA-COAD. Column names of the plot are the sample ID. **(B)** Difference in the proportions of 22 immune cell types between patients in the high-risk and low-risk groups. *p < 0.05; **p < 0.01; ***p < 0.001; and ****p < 0.0001; ns, not significant.

**FIGURE 10:**
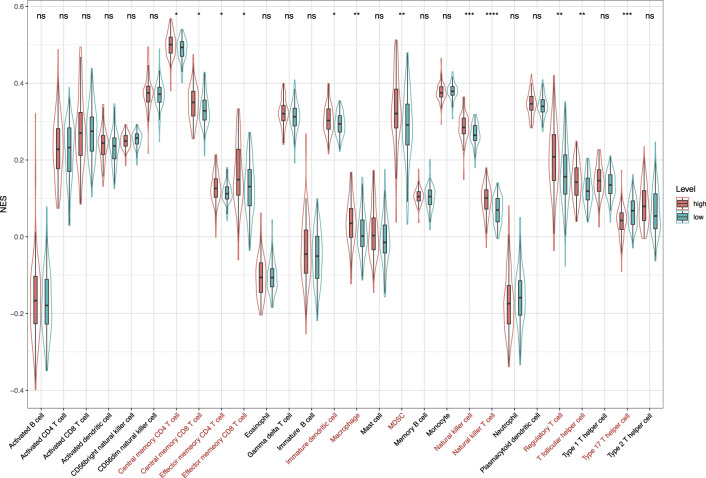
Normalized enrichment scores of 28 kinds of immune cells in the high-risk and low-risk groups. *p < 0.05; **p < 0.01; ***p < 0.001; and ****p < 0.0001; ns, not significant.

After the filtration of samples with p<0.05 *via* CIBERSORT, we obtained fractions of 22 immune cell types in 156 CC patients, including 74 patients in the high-risk group and 82 patients in the low-risk group. The relative fractions of 22 immune cell types are shown in Figure 9A. From Figure 9A, we can find that the highest proportion of patients in the high-risk group was macrophages M0 (24.3%), followed by macrophages M2 (12.9%) and mast cells activated (12.5%). Meanwhile, the highest proportion of patients in the low-risk group was macrophages M0 (17.2%), followed by mast cells activated (12.7%) and macrophages M2 (12.4%). As shown in Figure 9B, the distribution of six immune cell types had a significant difference between the high-risk and low-risk groups, which also exhibited higher infiltration of macrophages M0 and T cells regulatory, and lower infiltration of dendritic cells activated, NK cells activated, plasma cells, T cells CD4 memory activated, and T cells CD4 memory resting in the high-risk group. In addition, we also used the ssGSEA method to estimate the infiltration level of the 28 kinds of immune cells that were over-represented in the tumor microenvironment for the 156 CC patients. The results indicated that 12 kinds of immune cells had significant differences between the high-risk and low-risk groups (Figure 10). We also found that in addition to type 17 T helper cells, the other 11 kinds of immune cells (central memory CD4 T cells, central memory CD8 T cells, effector memory CD4 T cells, effector memory CD8 T cells, immature dendritic cells, macrophages, MDSC, natural killer cells, natural killer T cells, regulatory T cells, and T follicular helper cells) had a higher infiltration level in the high-risk group than in the low-risk group.

## Discussion

With the rapid development of next-generation sequencing technologies, computational tools are used to identify biomarkers and study cancer disease, which is an emergent field in cancer systems biology (Yang J. et al., 2020; Xu et al., 2020). CC is a high-incidence malignant tumor with a poor prognosis. Although targeted drugs can improve the prognosis of patients with CC, the mortality rate among patients remains high (Zhou and Ma, 2019). Therefore, reliable biomarkers must be identified for constructing a prognostic model to assess the prognosis and survival of CC patients.

Ferroptosis is morphologically, biochemically, and genetically distinct from other forms of cell death (Dixon et al., 2012). Previous studies have demonstrated that ferroptosis is involved in tumor immunization and cancer immunotherapy (Wang W. et al., 2019; Xu et al., 2021). Ferroptosis and iron metabolism play important roles in the pathogenesis of cancer. Moreover, ferroptosis-related lncRNA has also attracted attention (Mao et al., 2018; Wang M. et al., 2019; Yang Y. et al., 2020).

In this study, we constructed a prognostic model of three ferroptosis-related lncRNAs (XXbac-B476C20.9, TP73-AS1, and SNHG15) and showed that it had a good predictive ability for the overall survival of CC patients. Interestingly, literature mining revealed that three lncRNAs (XXbac-B476C20.9, TP73-AS1, and SNHG15) had been confirmed to be significantly associated with cancer. For example, the lncRNA XXbac-B476C20.9 was identified as a potential biomarker closely related to the prognosis of CC patients (Huang et al., 2019), which was consistent with our results. The overexpression of lncRNA TP73-AS1 was not only associated with metastasis and advanced clinical stages in colorectal cancer patients (Cai et al., 2018) but also promoted colorectal cancer cell migration and invasion (Li et al., 2019). Patients with high expression of lncRNA SNHG15 displayed a significantly shorter overall survival in COAD (Jiang et al., 2018). Moreover, the deregulation of the lncRNA SNHG15 strongly affected the proliferation, invasion, and tumor formation abilities of colorectal cancer cells (Saeinasab et al., 2019). The aforementioned previous studies further corroborated the results of our study.

We also investigated the underlying molecular mechanism by which the prognostic model is involved in the occurrence and development of CC through the GSEA analysis. Previous studies have also shown that GPI anchor biosynthesis, complement and coagulation cascades, and focal adhesion could play an important role in the progression of colorectal cancer (Cubiella et al., 2018; Gu et al., 2018; Xing et al., 2020). ECM receptor interaction, focal adhesion, and glycosaminoglycan biosynthesis chondroitin sulfate enriched in the high-risk group were related to cell motility, cell proliferation, and cell differentiation, which play a crucial role in the invasion of cancer cells (Han et al., 2021). Moreover, the Hallmark pathway analysis showed that epithelial-mesenchymal transition, apical junction, angiogenesis, and hedgehog signaling were enriched in the high-risk group, which was consistent with a previous study on CC (Yang et al., 2022). It was revealed that the mitotic spindle might lead to tumor formation in multiple tissues including colon cancer (Pussila et al., 2018). Bile acid metabolism was found to impact the microbial composition in colon cancer (Kennedy and Chang, 2020). Therefore, it is plausible that the prognostic model based on the three ferroptosis-related lncRNAs is highly correlated with CC.

Notably, our study found that the infiltration levels of macrophages M0, macrophages M2, and mast cells activated were significantly higher in the high-risk group. It has been shown that macrophages M0 were associated with the survival risk of CC, and the relative fraction of macrophages M0 was significantly increased in CC tissues compared with healthy bowel tissues (Wu et al., 2020). In addition, macrophages M2 induce the epithelial-mesenchymal transition phenotype in CC cells (Lee et al., 2020). The mast cells activated were C3-associated immune cells, where the C3 gene can predict the prognosis of colorectal adenocarcinoma (Liu and Wang, 2021). After analyzing the 28 kinds of immune cells that are over-represented in the tumor microenvironment, we also found that 12 kinds of immune infiltration cells are significantly different between the high-risk and low-risk groups, especially natural killer cells and natural killer T cells. El-Deeb et al. (2022) have found that the natural killer cells activated by the alginate/κ-carrageenan oral microcapsules lead to apoptosis in the colon cancer Caco-2 cells. Yoshioka et al. (2012) showed that the number of colon tumors and natural killer T cells significantly decreased in the mice in the treated group. In summary, the results indicated that the prognostic model was associated with immune infiltration of CC and might provide a reference for the immunotherapy of CC.

## Conclusion

In conclusion, we analyzed the lncRNA expression and clinical profiles in TCGA-COAD and GEO databases. Three differentially expressed ferroptosis-related lncRNAs (XXbac-B476C20.9, TP73-AS1, and SNHG15) were identified as biomarkers to establish a prognostic model for CC patients. The limitation to our study is that the prognostic model was constructed and validated on the database publicly available online. Future prospective clinical trials are required to further consolidate the effectiveness of the prognostic model.

## Data Availability

The original contributions presented in the study are included in the article/Supplementary Material; further inquiries can be directed to the corresponding author.
