# Supplementary material for: A Prognostic Ferroptosis-Related lncRNA Model Associated With Immune Infiltration in Colon Cancer
Source: Front Genet. 2022 Aug 31;13:934196. doi: 10.3389/fgene.2022.934196 (PMC9470855; doi:10.3389/fgene.2022.934196)
Supplement: Supplementary file 2 [file DataSheet1.docx]

Supplementary Material

# Supplementary Table

**Table S1** A total of 259 ferroptosis-related genes from the FerrDb database.

| Symbol | Name |
| --- | --- |
| RPL8 | Ribosomal protein L8 |
| IREB2 | Iron response element binding protein 2 |
| ATP5MC3 | ATP synthase membrane subunit c locus 3 |
| CS | Citrate synthase |
| EMC2 | ER membrane protein complex subunit 2 |
| ACSF2 | Acyl-CoA synthetase family member 2 |
| NOX1 | Nicotinamide adenine dinucleotide phosphate (NADPH) oxidase (NOX) 1 |
| CYBB | Cytochrome b-245 beta chain |
| NOX3 | Nicotinamide adenine dinucleotide phosphate (NADPH) oxidase (NOX) 3 |
| NOX4 | Nicotinamide adenine dinucleotide phosphate (NADPH) oxidase (NOX) 4 |
| NOX5 | Nicotinamide adenine dinucleotide phosphate (NADPH) oxidase (NOX) 5 |
| DUOX1 | Dual oxidase 1 |
| DUOX2 | Dual oxidase 2 |
| G6PD | Glucose-6-phosphate dehydrogenase |
| PGD | Phosphoglycerate dehydrogenase |
| PIK3CA | Phosphatidylinositol-4,5-bisphosphate 3-kinase catalytic subunit alpha |
| FLT3 | Fms related tyrosine kinase 3 |
| SCP2 | Sterol carrier protein 2 |
| TP53 | Tumor protein p53 |
| ACSL4 | Acyl-CoA synthetase long chain family member 4 |
| LPCAT3 | Lysophosphatidylcholine acyltransferase 3 |
| NRAS | NRAS proto-oncogene, GTPase |
| KRAS | KRAS proto-oncogene, GTPase |
| HRAS | HRas proto-oncogene, GTPase |
| TF | Transferrin |
| TFRC | Transferrin receptor |
| TFR2 | Transferrin receptor 2 |
| SLC38A1 | Solute carrier family 38 member 1 |
| SLC1A5 | Solute carrier family 1 member 5 |
| GLS2 | Glutaminase 2 |
| GOT1 | Glutamic-oxaloacetic transaminase 1 |
| CARS1 | Cysteinyl-tRNA synthetase 1 |
| ALOX5 | Arachidonate 5-lipoxygenase |
| KEAP1 | Kelch like ECH associated protein 1 |
| HMOX1 | Heme oxygenase 1 |
| ATG5 | Autophagy related 5 |
| ATG7 | Autophagy related 7 |
| NCOA4 | Nuclear receptor coactivator 4 |
| ALOX12 | Arachidonate 12-lipoxygenase, 12S type |
| ALOX12B | Arachidonate 12-lipoxygenase, 12R type |
| ALOX15 | Arachidonate 15-lipoxygenase |
| ALOX15B | Arachidonate 15-lipoxygenase type B |
| ALOXE3 | Arachidonate lipoxygenase 3 |
| PHKG2 | Phosphorylase kinase catalytic subunit gamma 2 |
| ACO1 | Aconitase 1 |
| G6PDX | _NA_ |
| ULK1 | Unc-51 like autophagy activating kinase 1 |
| ATG3 | Autophagy related 3 |
| ATG4D | Autophagy related 4D cysteine peptidase |
| BECN1 | Beclin 1 |
| MAP1LC3A | Microtubule associated protein 1 light chain 3 alpha |
| GABARAPL2 | GABA type A receptor associated protein like 2 |
| GABARAPL1 | GABA type A receptor associated protein like 1 |
| ATG16L1 | Autophagy related 16 like 1 |
| WIPI1 | WD repeat domain, phosphoinositide interacting 1 |
| WIPI2 | WD repeat domain, phosphoinositide interacting 2 |
| SNX4 | Sorting nexin 4 |
| ATG13 | Autophagy related 13 |
| ULK2 | Unc-51 like autophagy activating kinase 2 |
| SAT1 | Spermidine/spermine N1-acetyltransferase 1 |
| EGFR | Epidermal growth factor receptor |
| MAPK3 | Mitogen-activated protein kinase 3 |
| MAPK1 | Mitogen-activated protein kinase 1 |
| BID | BH3 interacting domain death agonist |
| ZEB1 | Zinc finger E-box binding homeobox 1 |
| DPP4 | Dipeptidyl peptidase 4 |
| CDKN2A | Cyclin dependent kinase inhibitor 2A |
| PEBP1 | Phosphatidylethanolamine binding protein 1 |
| SOCS1 | Suppressor of cytokine signaling 1 |
| CDO1 | Cysteine dioxygenase type 1 |
| MYB | MYB proto-oncogene, transcription factor |
| MAPK8 | Mitogen-activated protein kinase 8 |
| MAPK9 | Mitogen-activated protein kinase 9 |
| CHAC1 | ChaC glutathione specific gamma-glutamylcyclotransferase 1 |
| MAPK14 | Mitogen-activated protein kinase 14 |
| LINC00472 | Long intergenic non-protein coding RNA 472 |
| PRKAA2 | Protein kinase AMP-activated catalytic subunit alpha 2 |
| PRKAA1 | Protein kinase AMP-activated catalytic subunit alpha 1 |
| ELAVL1 | ELAV like RNA binding protein 1 |
| BAP1 | BRCA1 associated protein 1 |
| ABCC1 | ATP binding cassette subfamily C member 1 |
| MIR6852 | microRNA 6852 |
| ACVR1B | Activin A receptor type 1B |
| TGFBR1 | Transforming growth factor beta receptor 1 |
| EPAS1 | Endothelial PAS domain protein 1 |
| HILPDA | Hypoxia inducible lipid droplet associated |
| HIF1A | Hypoxia inducible factor 1 subunit alpha |
| IFNG | Interferon gamma |
| ANO6 | Anoctamin 6 |
| LPIN1 | Lipin 1 |
| HMGB1 | High mobility group box 1 |
| TNFAIP3 | TNF alpha induced protein 3 |
| TLR4 | Toll like receptor 4 |
| ATF3 | Activating transcription factor 3 |
| ATM | ATM serine/threonine kinase |
| YY1AP1 | YY1 associated protein 1 |
| EGLN2 | Egl-9 family hypoxia inducible factor 2 |
| MIOX | Myo-inositol oxygenase |
| TAZ | Tafazzin |
| MTDH | Metadherin |
| IDH1 | Isocitrate dehydrogenase (NADP(+)) 1 |
| SIRT1 | Sirtuin 1 |
| FBXW7 | F-box and WD repeat domain containing 7 |
| PANX1 | Pannexin 1 |
| DNAJB6 | DnaJ heat shock protein family (Hsp40) member B6 |
| BACH1 | BTB domain and CNC homolog 1 |
| LONP1 | Lon peptidase 1, mitochondrial |
| PTGS2 | Prostaglandin-endoperoxide synthase 2 |
| DUSP1 | Dual specificity phosphatase 1 |
| NOS2 | Nitric oxide synthase 2 |
| NCF2 | Neutrophil cytosolic factor 2 |
| MT3 | Metallothionein 3 |
| UBC | Ubiquitin C |
| ALB | Albumin |
| TXNRD1 | Thioredoxin reductase 1 |
| SRXN1 | Sulfiredoxin 1 |
| GPX2 | Glutathione peroxidase 2 |
| BNIP3 | BCL2 interacting protein 3 |
| OXSR1 | Oxidative stress responsive kinase 1 |
| SELENOS | Selenoprotein S |
| ANGPTL7 | Angiopoietin like 7 |
| SLC7A11 | Solute carrier family 7 member 11 |
| DDIT4 | DNA damage inducible transcript 4 |
| LOC284561 | _NA_ |
| ASNS | Asparagine synthetase (glutamine-hydrolyzing) |
| TSC22D3 | TSC22 domain family member 3 |
| DDIT3 | DNA damage inducible transcript 3 |
| JDP2 | Jun dimerization protein 2 |
| SESN2 | Sestrin 2 |
| SLC1A4 | Solute carrier family 1 member 4 |
| PCK2 | Phosphoenolpyruvate carboxykinase 2, mitochondrial |
| TXNIP | Thioredoxin interacting protein |
| VLDLR | Very low density lipoprotein receptor |
| GPT2 | Glutamic--pyruvic transaminase 2 |
| PSAT1 | Phosphoserine aminotransferase 1 |
| LURAP1L | Leucine rich adaptor protein 1 like |
| SLC7A5 | Solute carrier family 7 member 5 |
| HERPUD1 | Homocysteine inducible ER protein with ubiquitin like domain 1 |
| XBP1 | X-box binding protein 1 |
| SLC3A2 | Solute carrier family 3 member 2 |
| CBS | Cystathionine beta-synthase |
| ATF4 | Activating transcription factor 4 |
| ZNF419 | Zinc finger protein 419 |
| KLHL24 | Kelch like family member 24 |
| TRIB3 | Tribbles pseudokinase 3 |
| ZFP69B | ZFP69 zinc finger protein B |
| ATP6V1G2 | ATPase H+ transporting V1 subunit G2 |
| VEGFA | Vascular endothelial growth factor A |
| GDF15 | Growth differentiation factor 15 |
| TUBE1 | Tubulin epsilon 1 |
| ARRDC3 | Arrestin domain containing 3 |
| CEBPG | CCAAT enhancer binding protein gamma |
| SNORA16A | Small nucleolar RNA, H/ACA box 16A |
| RGS4 | Regulator of G protein signaling 4 |
| BLOC1S5-TXNDC5 | BLOC1S5-TXNDC5 readthrough (NMD candidate) |
| LOC390705 | _NA_ |
| EIF2S1 | Eukaryotic translation initiation factor 2 subunit 1 |
| KIM-1 | Kidney injury molecule-1 |
| IL6 | Interleukin 6 |
| CXCL2 | C-X-C motif chemokine ligand 2 |
| RELA | RELA proto-oncogene, NF-kB subunit |
| HSD17B11 | Hydroxysteroid 17-beta dehydrogenase 11 |
| AGPAT3 | 1-acylglycerol-3-phosphate O-acyltransferase 3 |
| SETD1B | SET domain containing 1B, histone lysine methyltransferase |
| FTL | Ferritin light chain |
| MAFG | MAF bZIP transcription factor G |
| IL33 | Interleukin 33 |
| FTH1 | Ferritin heavy chain 1 |
| SLC40A1 | Solute carrier family 40 member 1 |
| GPX4 | Glutathione peroxidase 4 |
| HAMP | Hepcidin antimicrobial peptide |
| HSPB1 | Heat shock protein family B (small) member 1 |
| NFE2L2 | Nuclear factor, erythroid 2 like 2 |
| STEAP3 | STEAP3 metalloreductase |
| DRD5 | Dopamine receptor D5 |
| DRD4 | Dopamine receptor D4 |
| MAP3K5 | Mitogen-activated protein kinase kinase kinase 5 |
| SLC2A1 | Solute carrier family 2 member 1 |
| SLC2A3 | Solute carrier family 2 member 3 |
| SLC2A6 | Solute carrier family 2 member 6 |
| SLC2A8 | Solute carrier family 2 member 8 |
| SLC2A12 | Solute carrier family 2 member 12 |
| GLUT13 | _NA_ |
| SLC2A14 | Solute carrier family 2 member 14 |
| EIF2AK4 | Eukaryotic translation initiation factor 2 alpha kinase 4 |
| TFAP2C | Transcription factor AP-2 gamma |
| SP1 | Sp1 transcription factor |
| HBA1 | Hemoglobin subunit alpha 1 |
| NNMT | Nicotinamide N-methyltransferase |
| PLIN4 | Perilipin 4 |
| HIC1 | HIC ZBTB transcriptional repressor 1 |
| STMN1 | Stathmin 1 |
| RRM2 | Ribonucleotide reductase regulatory subunit M2 |
| CAPG | Capping actin protein, gelsolin like |
| HNF4A | Hepatocyte nuclear factor 4 alpha |
| NGB | Neuroglobin |
| YWHAE | Tyrosine 3-monooxygenase/tryptophan 5-monooxygenase activation protein epsilon |
| GABPB1 | GA binding protein transcription factor subunit beta 1 |
| AURKA | Aurora kinase A |
| MIR4715 | microRNA 4715 |
| RIPK1 | Receptor interacting serine/threonine kinase 1 |
| PRDX1 | Peroxiredoxin 1 |
| MIR30B | microRNA 30b |
| AKR1C1 | Aldo-keto reductase family 1 member C1 |
| AKR1C2 | Aldo-keto reductase family 1 member C2 |
| AKR1C3 | Aldo-keto reductase family 1 member C3 |
| RB1 | RB transcriptional corepressor 1 |
| HSF1 | Heat shock transcription factor 1 |
| GCLC | Glutamate-cysteine ligase catalytic subunit |
| SQSTM1 | Sequestosome 1 |
| NQO1 | NAD(P)H quinone dehydrogenase 1 |
| MUC1 | Mucin 1, cell surface associated |
| MT1G | Metallothionein 1G |
| CISD1 | CDGSH iron sulfur domain 1 |
| FANCD2 | FA complementation group D2 |
| FTMT | Ferritin mitochondrial |
| HSPA5 | Heat shock protein family A (Hsp70) member 5 |
| HELLS | Helicase, lymphoid specific |
| SCD | Stearoyl-CoA desaturase |
| FADS2 | Fatty acid desaturase 2 |
| SRC | SRC proto-oncogene, non-receptor tyrosine kinase |
| STAT3 | Signal transducer and activator of transcription 3 |
| PML | Promyelocytic leukemia |
| MTOR | Mechanistic target of rapamycin kinase |
| NFS1 | NFS1 cysteine desulfurase |
| TP63 | Tumor protein p63 |
| CDKN1A | Cyclin dependent kinase inhibitor 1A |
| MIR137 | microRNA 137 |
| ENPP2 | Ectonucleotide pyrophosphatase/phosphodiesterase 2 |
| VDAC2 | Voltage dependent anion channel 2 |
| FH | Fumarate hydratase |
| CISD2 | CDGSH iron sulfur domain 2 |
| MIR9-1 | microRNA 9-1 |
| MIR9-2 | microRNA 9-2 |
| MIR9-3 | microRNA 9-3 |
| ISCU | Iron-sulfur cluster assembly enzyme |
| ACSL3 | Acyl-CoA synthetase long chain family member 3 |
| OTUB1 | OTU deubiquitinase, ubiquitin aldehyde binding 1 |
| CD44 | CD44 molecule (Indian blood group) |
| LINC00336 | Long intergenic non-protein coding RNA 336 |
| BRD4 | Bromodomain containing 4 |
| PRDX6 | Peroxiredoxin 6 |
| MIR17 | microRNA 17 |
| NF2 | Neurofibromin 2 |
| ARNTL | Aryl hydrocarbon receptor nuclear translocator like |
| JUN | Jun proto-oncogene, AP-1 transcription factor subunit |
| CA9 | Carbonic anhydrase 9 |
| TMBIM4 | Transmembrane BAX inhibitor motif containing 4 |
| PLIN2 | Perilipin 2 |
| MIR212 | microRNA 212 |
| Fer1HCH | Ferritin 1 Heavy Chain Homolog |
| AIFM2 | Apoptosis inducing factor mitochondria associated 2 |
| LAMP2 | Lysosomal associated membrane protein 2 |
| ZFP36 | ZFP36 ring finger protein |
| PROM2 | Prominin 2 |
| CHMP5 | Charged multivesicular body protein 5 |
| CHMP6 | Charged multivesicular body protein 6 |
| CAV1 | Caveolin 1 |
| GCH1 | GTP cyclohydrolase 1 |

**Table S2**: Univariate and multivariate Cox regression analysis in the training set, test set, and whole set.

| Variables | Univariate analysis | | | | | Multivariate analysis | | | | |
| --- | --- | --- | --- | --- | --- | --- | --- | --- | --- | --- |
|  | HR | 95% CI | P value | | | HR | 95% CI | | P value | |
| **Training set (n= 185)** |  |  | |  |  | | |  | |  |
| ^a^Risk score | 2.7183 | 1.4594−5.0631 | | 0.0016 | 2.2628 | | | 1.2171−4.2071 | | 0.0099 |
| Age (<70 vs. ≥70) | 1.7786 | 0.8676−3.6463 | | 0.1159 | - | | | - | | - |
| Gender (male vs. female) | 0.691 | 0.3431−1.3918 | | 0.3009 | - | | | - | | - |
| T stage (T1+T2 vs. T3 +T4) | 5.6985 | 0.7731−42.0014 | | 0.0877 | - | | | - | | - |
| N stage (N0+N1 vs. N2 +N3) | 3.4704 | 1.6929−7.1145 | | 0.0007 | 1.3148 | | | 0.5235−3.3026 | | 0.5603 |
| M stage (M0 vs. M1) | 5.651 | 2.7134−11.7688 | | <0.0001 | 3.541 | | | 1.4219−8.8181 | | 0.0066 |
| AJCC Stage (I+II vs. III+IV) | 3.0344 | 1.4811−6.217 | | 0.0024 | 1.3472 | | | 0.4818−3.7672 | | 0.57 |
| **Test set (n=185)** |  |  | |  |  | | |  | |  |
| ^a^Risk score | 1.7131 | 1.2905−2.274 | | 0.0002 | 1.82 | | | 1.3172−2.5147 | | 0.0003 |
| Age (<70 vs. ≥70) | 1.5814 | 0.8685−2.8795 | | 0.1339 | - | | | - | | - |
| Gender (male vs. female) | 1.3901 | 0.7547−2.5603 | | 0.2906 | - | | | - | | - |
| T stage (T1+T2 vs. T3 +T4) | 10.5809 | 1.455−76.9442 | | 0.0198 | 3.8149 | | | 0.4838−30.0802 | | 0.2038 |
| N stage (N0+N1 vs. N2 +N3) | 4.2915 | 2.3286−7.9091 | | <0.0001 | 1.8939 | | | 0.9654−3.7152 | | 0.0632 |
| M stage (M0 vs. M1) | 4.6458 | 2.5328−8.5217 | | <0.0001 | 2.6663 | | | 1.3578−5.2358 | | 0.0044 |
| AJCC Stage (I+II vs. III+IV) | 4.7581 | 2.2837−9.9137 | | <0.0001 | 2.1354 | | | 0.8468−5.3848 | | 0.1079 |
| **Whole set (n=370)** |  |  | |  |  | | |  | |  |
| ^a^Risk score | 1.9098 | 1.4818−2.4614 | | <0.0001 | 1.8011 | | | 1.3437−2.4143 | | <0.0001 |
| Age (<70 vs. ≥70) | 1.6432 | 1.0436−2.5872 | | 0.032 | 1.8217 | | | 1.129−2.9395 | | 0.014 |
| Gender (male vs. female) | 1.0321 | 0.6564−1.6229 | | 0.891 | - | | | - | | - |
| T stage (T1+T2 vs. T3 +T4) | 8.0067 | 1.9615−32.6834 | | 0.0037 | 3.4588 | | | 0.8143−14.6918 | | 0.0927 |
| N stage (N0+N1 vs. N2 +N3) | 3.9094 | 2.4619−6.2078 | | <0.0001 | 1.3387 | | | 0.7694−2.3292 | | 0.3019 |
| M stage (M0 vs. M1) | 5.1324 | 3.2187−8.1838 | | <0.0001 | 2.8319 | | | 1.6436−4.8792 | | 0.0002 |
| AJCC Stage (I+II vs. III+IV) | 3.9042 | 2.3603−6.4582 | | <0.0001 | 2.1165 | | | 1.0786−4.1532 | | 0.0293 |

**Notes.** ^a^Derived from the proposed risk score model
